# Dilatation of Common Iliac Arteries after Endovascular Infrarenal Abdominal Aortic Repair with Bell-Bottom Extension

**DOI:** 10.5935/1678-9741.20160032

**Published:** 2016

**Authors:** Gustavo José Politzer Telles, Álvaro Razuk Filho, Walter Khegan Karakhanian, Paulo Fernandes Saad, Karen Ruggeri Saad, Jong Hun Park, Leticia Cristina Dalledone Siqueira, Roberto Augusto Caffaro

**Affiliations:** 1 Faculdade de Ciências Médicas da Santa Casa de São Paulo (FCMSCSP), São Paulo, SP, Brazil.; 2 Universidade Federal do Vale do São Francisco (UNIVASF), Petrolina, PE, Brazil.

**Keywords:** Endovascular Procedures, Aortic Aneurysm, Iliac Aneurysm, Endoleak

## Abstract

**Objective:**

Endovascular techniques to treat abdominal aortic aneurysms results in lower morbidity and mortality rates. However, dilation of the common iliac arteries prevents adequate distal sealing, which compromises the procedure success. The aim of this study is report the long-term outcomes of patients with abdominal aortic aneurysms associated with aneurysm of the common iliac artery following endovascular repair using a bifurcated bell-bottom stent graft.

**Methods:**

This is a retrospective study that evaluated patients treated with bifurcated bell-bottom extension stent grafts to repair an infrarenal abdominal aortic aneurysm and who had at least one common iliac artery with dilatation > 1.5 cm for at least 12 months after the endovascular intervention.

**Results:**

Thirty-eight patients with a mean age of 70.4±8.2 years were included. Stent graft placement was followed by dilation of the common iliac artery aneurysms in 35.3% of cases; endoleak and reoperation rates were 17.6% and 15.7%, respectively. Younger patients showed a higher rate of artery diameter increase following the procedure. The average arterial dilation was 16% in the first year, 29% in the second year, 57% in the third year and 95% from the fourth year until the end of follow-up.

**Conclusion:**

Repair of infrarenal abdominal aortic aneurysms with bifurcated bell-bottom type stents when there is common iliac artery dilation is a good therapeutic option to preserve hypogastric flow. The rate of endoleak was 17.6%, and 15.7% of cases required reoperation. Younger patients are more likely to experience dilation of the common iliac artery after the procedure.

**Table:** 

Abbreviations, acronyms & symbols	
AAAs	= Abdominal aortic aneurysms
CIAs	= Common iliac arteries
CT	= Computed tomography

## INTRODUCTION

Aneurysms of the common iliac arteries (CIAs) are observed in about 20-25% of all cases of abdominal aortic aneurysms (AAAs), whereas the occurrence of isolated CIA aneurysms is very rare^[1]^.

The literature has shown that use of endovascular techniques to treat AAAs results in lower intraoperative and short-term morbidity and mortality rates. However, dilation of one or both CIAs prevents adequate distal sealing, which compromises the success of the procedure^[2,3]^.

Although treatment of CIA aneurysms that coexist with AAAs has not yet been standardized^[3]^, several endovascular techniques are available, which can be divided into those that either sacrifice or preserve hypogastric flow^[4-7]^. The techniques that sacrifice hypogastric flow have a disadvantage in that they can cause buttock claudication, sexual dysfunction, and ischemic colitis^[8-11]^.

Of the various techniques that preserve hypogastric flow, the bell-bottom technique is commonly used^[12,13]^. This consists of placing a bifurcated stent graft extension as far as the bifurcation of the CIA; this extension has a larger diameter in its terminal section so as to promote proper coaptation with the wall of the dilated artery. Thus, hypogastric flow can be preserved and complications from ischemia can be avoided^[12]^. This technique requires a shorter operation time and is associated with a lower rate of complications compared with other techniques^[13]^; however, more data are needed to evaluate the long-term outcomes of this procedure to treat CIA aneurysms in patients with AAAs. The aim of this study was to analyze the occurrence of endoleak, dilation, and the need for reoperation during a oneyear follow-up period after the procedure.

## METHODS

This is a retrospective study with a convenience sample whose was approved by the Ethics Committee of our institution (006/10).

Patients who were treated with bifurcated bell-bottom stent grafts to repair an infrarenal AAA of atherosclerotic etiology that had > 1.5 cm dilation of at least one CIA were included. The patients were monitored for at least 12 months after the endovascular intervention.

All patients were referred for the proposed treatment based on the diameter of the AAA, and all underwent the same surgical technique^[12,13]^. The CIA dilation was treated by inserting bell-bottom extensions measuring 16 mm, 18 mm, 20 mm, 22 mm, or 24 mm, according to the size of the treated artery.

The diameters of the AAA and right and left CIAs were measured from computed tomography (CT) scan performed before and at the time of the procedure. CT scans were also performed at follow-up visits to measure the diameter of the aneurysm and document any endoleaks and the need for reoperation. These visits took place one, six, and twelve months after the procedure. After one year, patients were monitored with ultrasound every six months. In cases where the ultrasound examination showed dilation of the aneurysm or endoleaks, a new CT scan was obtained^[14]^.

Data on the number of comorbidities and the presence of systemic arterial hypertension, heart disease, cancer, chronic renal failure, dyslipidemia, and peripheral vascular disease and diabetes mellitus were also collected.

Endoleaks were classified according to their origin. In type I, the anchor points may be proximal (IA) or distal (IB). Type II are caused by refilling of the aneurysmal sac from the aortic collateral vessels. Type III originate from a partial or complete decoupling or fractures of components of the modulated stent and cause persistent flow within the aneurysmal sack. Type IV endoleaks are associated with porosity of the endograft.

The statistics used included analysis of variance (ANOVA) to compare ≥ three groups, and Student's t test or chi-square test to compare two groups. Spearman's rank correlation coefficient was used to establish possible correlations between these age, comorbidities, and artery diameter before treatment. The correlation coefficient was classified as: strong (> 0.75), medium (> 0.5), or low (<0.5). A significance level of 5% (*P*<0.05) was adopted for all statistical tests.

## RESULTS

Thirty-eight patients (34 men and 4 women) with a mean age of 70.4±8.2 years had bifurcated bell-bottom stent graft placement to treat an infrarenal AAA associated with dilation of at least one CIA. Eight procedures were performed on the right iliac artery, 17 on the left iliac artery, and 13 on both of the CIAs. Therefore, 21 procedures were analyzed for the right iliac artery and 30 for the left iliac artery. The average diameter of the treated arteries was 2.1±0.4 cm, ranging from 1.5 cm to 3.2 cm and the distribution of sizes of stent-grafts used was: 12% of 16 mm, 39% of 18 mm, 29% of 20 mm, 12% of 22 mm and 8% of 24 mm.

The stent-grafts used were Talent (66%), Zenith (18%), Apollo (13%) and Anaconda (3%). There were no thrombus or significant calcification in the distal landing zone of bell-bottom extension.

**Fig. 1:**
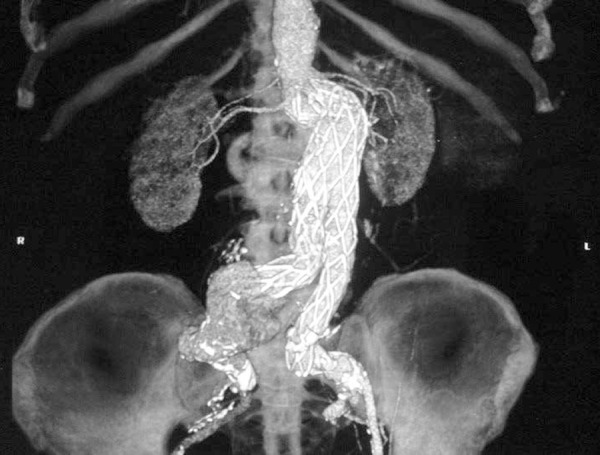
Computed tomography of the patient number 18, showing dilatation of the artery with right iliac with type IB endoleak after treatment with bifurcated stent-graft with a bell-bottom extension.

Endoleaks were observed in 9 CIAs (17.6%), consisting of 6 of the 30 left iliac arteries (20%) and 3 of the 21 right arteries (14.3%; Figure 1). There was a statistically similar distribution in frequency of endoleaks between the two arteries (x^2^=0.02;* P*=0.887). The characteristics of patients who had endoleak and/or need to be re-operated are shown in Table 1, including the five cases (55.6%) that required reoperation.

**Table 1:** Characteristics of patients who had endoleak and/or need to be re-operated.

Initials	Sex	Age (years)	Comorbidities	AAA Diameter	CIA Diameter	Endoprosthesis	Follow-up (months)	Endoleak	Reoperation
LM	M	74	SAH+HD+PVD+LD	6	2.5 (L)	Talent	76	II	
JLPO	M	73	EX-TB	6.8	2.0 (L)	Apolo	57	IB	Yes
					3.2 (R)			IB	Yes
FV	M	70		5.5	1.9 (R)	Talent	29	II	
LAC	M	60	TB+HD+LD	7.0	2.0 (L)	Talent	49		Yes
CR	M	52	EX-TB+SAH+HD	6.4	2.1 (R)	Apolo	36	IB	Yes
CN	M	72	SAH+DM+CRF	6.0	2.5 (R)	Apolo	22		Yes
DFM	M	72	EX-TAB+SAH+PVD+C	6.5	2.8 (L)	Talent	14	II	Yes
JST	M	61	SAH	5.2	2.0 (R)	Talent	26		Yes
JBN	M	90	SAH	7.5	2.3 (L)	Zenith	29	II	
EE	M	65	SAH+HD+COPD	6.0	2.0 (L)	Zenith	12	IB	Yes
SV	M	65	EX-TB	9.5	2.6 (L)	Zenith	13	II	

M=male; AAA=abdominal aortic aneurysms; CIA= aneurysms of the common iliac arteries; SAH=systemic arterial hypertension; HD=heart disease; C=cancer; CRF=chronic renal failure; PVD=peripheral vascular disease; DM=diabetes mellitus; TB=tabagist; EX-TB= ex-tabagist; LD=liver disease; COPD=chronic obstructive pulmonary disease; R=right; L=left

There was a need to reoperate in eight cases (15.7%), three of which had no leak on imaging but showed a significant increase in the diameter of the CIA. Four of the reoperations were performed on right iliac arteries (50%) and the other four on left iliac arteries (50%). No reoperation was necessary in 84.3% of the cases over a median follow-up of 25.8 months. None of the patients required additional conventional surgical procedure.

Table 2 shows the difference in mean age and the number of comorbidities between the patients with and without endoleaks. Patients who required reoperation had a significantly lower mean age, and higher mean number of comorbidities compared with those who did not require reoperation.

**Table 2:** Mean and standard deviations for age and number of comorbidities of patients evolving with and without endoleaks and those with and without reoperation.

	Endoleak		Reoperation	
	Yes	No	Yes	No
Age	70.1±10.7	70.5±7.6	65.0±7.8	71.6±7.8
Number of comorbidities	2.4±1.3	2.0±1.0	2.9±1.1	1.9±1.0

The mean time between treatment and the last CT scan for all patients was 25.8±14.9 months, and there no differences in this time between the group of patients with good outcome and the group with complications that included endoleaks, reoperation or both (Table 3). Furthermore, no difference in this interval was found in patients who required a reoperation compared with those who did not require a reoperation (36.8±23.1 and 23.7±12.2 months, respectively; *P*=0.160), or between cases with or without endoleaks (38.1±26.1 and 23.1±9.9 months, respectively; *P*=0.126).

**Table 3:** Means and standard deviations in time (months) elapsed between treatment and findings on last CT scan.

Without complications	With complications	Complications		
		Endoleak	Reoperation	Endoleak + Reoperation
22.4±9.4	36.5±23.3	36.0±27.1	31.7+14.2	39.5±28.3

*P*=0.063    ANOVA: *P*>0.05

The distal diameter of the device used (bell bottom) showed no correlation with the outcome of the procedures (r=0.189). Although the mean diameter in procedures with good outcomes (19.0±2.2 mm) tended (*P*=0.074) to be lower than that in patients who developed endoleaks and/or required reoperation (20.4±1.9 mm), the distribution of frequencies of procedures with good outcomes and those with complications according to device diameter showed that diameter did not influence outcome. Likewise, no difference (*P*>0.05) was seen in the mean diameter of the devices used when comparing cases that had endoleaks (20.5±2.5 mm), with those that required reoperation (21.3±1.1 mm) or those that had both of these events (19.5±1.9 mm).

Analysis of the last CT scans showed that in 33 cases (64.7%) the diameter of the CIAs repaired remained either unchanged or was reduced by up to 20% compared with the diameter obtained by CT scan prior to treatment. In the remaining 18 cases (35.3%), an increase in the diameter of the repaired arteries was found, ranging from 4% to 168% (mean, 48%±26%).

When patients were stratified into two groups based on an enlargement of the repaired artery on the last CT scan (Table 4), there were no differences in the mean follow-up period or artery diameter before treatment between these two groups. Again, the age of patients who had enlargement of the repaired artery on the last CT scan was significantly lower than the age of patients who did not have dilation. The mean percentage dilation of the arteries was 16% in the first year, 29% in the second year, 57% in the third year, and 95% from the fourth year until the end of follow-up.

**Table 4:** Means and standard deviations for patient age, follow-up time, and artery diameter before treatment, in cases with dilation of repaired artery on last CT scan.

Change in diameter of repaired artery	Patient age (years)	Follow-up time (months)	Artery diameter before treatment (cm)
None or reduction of up to 20%	72.0±7.1	25.7±15.4	2.2±0.4
Increase of between 4% and 168%	67.4±9.2	25.8±14.5	2.1±0.3
*P* value	0.047	0.981	0.481

## DISCUSSION

There is currently no standardized treatment for CIAs, whether isolated or associated with AAAs. However, endovascular techniques that preserve hypogastric flow are associated with a reduced frequency of complications, such as buttock claudication, sexual dysfunction, and ischemic colitis^[15]^.

Placement of a bell-bottom stent graft is one of the endovascular techniques that preserves hypogastric flow^[12]^ and was initially used to treat CIAs with diameters ranging from 1.5 cm to 2.4 cm. This technique has been used for a decade now, even in arteries with large diameters, and the results have compared favorably with other techniques^[5,13,16,17]^. However, there are few reports on the medium- and long-term outcomes of this endovascular procedure in patients with CIA aneurysms associated with AAAs.

This study evaluated the results of 51 endovascular procedures using the bell-bottom technique to repair CIA aneurysms associated with AAAs in 38 patients. The patients were predominantly men (89%), with a mean age of 70.4±8.2 years, a mean of 2.1±1.1 risk factors, and a median follow-up of 25.8 months.

In this sample of CIA aneurysms associated with AAAs, left common iliac artery (58.8%) was involved more often, whereas both CIAs had aneurysms in 13 patients (34.2%). A similar prevalence of bilateral involvement (32.2%) was also observed by Parlani et al.^[18]^ in a sample of 59 patients.

England et al.^[19]^ evaluated the outcome of CIA aneurysms treated using this technique over a median follow-up period of 24 months (range, 1-84 months). They compared 87 arteries with a diameter of < 1.8 cm and 30 arteries ranging from 1.8-2.5 cm in diameter. The results were statistically similar for both groups of patients with respect to endoleaks following the procedure. The authors reported three cases of type IB endoleaks (2.6%); the one-year reoperation rate was 8% for the smaller-diameter arteries and 16% for the larger-diameter arteries. They suggested that treatment of dilated CIAs with a diameter > 1.8 cm is associated with an increased risk of intervention. In our study, despite a similar average follow-up period, the results showed a higher overall rate of endoleaks (17.6%) and the need for reoperation (15.7% of procedures) than in their study, with 1.9% of reoperations occurring in the first year after treatment, 3.9% in the second year, 3.9% in the third, and 5.9% after more than three years. Despite the higher rate of complications, we observed no association between a greater initial artery diameter and endoleaks and/or the need for reoperation.

Our results also showed higher rates of type IB (7.8%) and II (9.8%) endoleaks, and a 15.7% rate of reoperation when compared with the results of Torsello et al.^[16]^, who reported a 3.4% and 2.2% rate of late-onset type IB and type II endoleaks, respectively. In that study, both endoleak types were accompanied by artery dilation in patients whose age and initial arterial diameter were similar to those in our study. For Torsello et al.^[16]^ , the reoperation rate at five years was 8.4%. However, Adiseshiah et al.^[20]^ argued that type IB endoleak rates would be more frequent with this type of endovascular treatment than those reported by Torsello et al.^[16]^, implying that the lower complication rates they observed could be attributed to the shorter follow-up time. The same factor may also explain the higher rate of endoleaks and the need for reoperation in our study, since our patient follow-up period was considerably longer.

Kirkwood et al.^[21]^ evaluated the results of endovascular treatment of CIAs during AAA repair performed in 671 patients at various centers, and monitored the patients annually for five years. They subdivided the study population according to the maximum pretreatment diameter of the iliac artery (> 2.0 cm and < 2.0 cm). The authors observed that iliac artery dilation after treatment was not different between these two subgroups, so that the initial diameter did not seem to affect postprocedure dilation. However, adverse events of greater severity occurred more frequently in patients whose arteries dilated after treatment, regardless of the initial diameter of the iliac artery involved. Dias et al.^[15]^ treated AAAs with branched stent grafts to preserve hypogastric flow and reported a reoperation rate of 18% after a mean follow-up period of 20 months. We note that there is considerable variation in the endoleak and reoperation rates reported by different authors. However, as we found in this study, most authors report that there was no significant difference when comparing groups stratified on the basis of the initial aneurysm diameter and endovascular technique used. This variation is probably due to the lack of a consistent follow-up period, along with differences in patient's baseline characteristics such as age and the number of risk factors.

In our study, endoleak rates were not associated with the mean age of patients. In contrast, reoperation rates were significantly more frequent in younger patients (mean of 65 years) and in those with a greater number of risk factors (mean of 2.9 risk factors). Furthermore, we found that patients with three associated risk factors had a significantly lower mean age (*P*=0.022) than those with one or two risk factors (*P*=0.050). We should first consider that older patients with more associated risk factors may not have had the opportunity to receive this type of treatment due to the problem of survival. Another possibility is that younger patients had longer follow-up; however, there was no significant correlation between patient age and follow-up time.

Patients who required reoperation were monitored for an average of 36.8±23.1 months, which was statistically similar to the follow-up time of patients with a favorable outcome (23.7±12.2 months). In the case of endoleaks, whether they were re-operated or not, no significant difference in follow-up time was observed between the patients with our without endoleaks; however, it is important to recognize that the follow-up time in cases with endoleaks was significantly greater than the follow-up time in cases with good outcomes (38.1±26.1 *vs.* 23.1±9.9 months, *P*<0.001).

According to Adiseshiah et al.^[20]^, type IB endoleaks are more common with endovascular treatment using the bell-bottom technique in patients who are monitored long-term (*i.e.*, with follow-up periods of > 5 years). In our study, out of the four cases with type IB endoleaks, two were observed at 11 and 14 months postoperative, and the other two were recorded at 69 months post-treatment and reflect a single patient who had bilateral involvement of their CIAs. Similarly, considering all types of endoleaks observed in this series, there was one case in the first year, two in the second year, three in the third year, and another three after approximately six years; overall, 66.7% of endoleaks were observed within the first three years following treatment. Furthermore, when analyzing endoleaks and reoperations as a subgroup of complications, we noted a significant, albeit weak, positive correlation (r=0.40) between follow-up time and the occurrence of such complications.

In 2001, Sahgal et al.^[22]^ analyzed changes in the diameter of 35 isolated CIA aneurysms with a follow-up period ranging from 13 to 72 months after endovascular treatment. The mean diameter of these aneurysms before treatment was 4.6±1.6 cm, and 94.3% had a mean reduction of 1.1±0.6 cm at a mean follow-up time of 31 months. The mean reduction in the first year of follow-up was 0.5 cm. The cases where aneurysm diameter increased, at 18 and 24 months post-treatment, were initially larger than 5 cm and evolved toward rupture.

In 64.7% of our cases, the treated arteries had the same diameter or experienced a 20% reduction compared with the initial diameter, and these changes were not related to followup time, pretreatment arterial diameter, or patient age. In the remaining cases (35.3%), we noted an increase in the diameter of the treated arteries ranging from 4% to 168% (mean, 48±26%) compared with pretreatment. In these cases, the diameter increase averaged 16% in the first year, 29% in the second year, 57% in the third year, and 95% from the fourth year until the end of follow-up. The increase in aneurysm diameter showed a significant positive correlation with follow-up time, and a negative correlation with patient age. It should be noted that all cases of reoperation involved dilation > 40% compared with the pretreatment diameter.

### Limitations

The design of this study does not allow the evaluation of the impact of bell-bottom stent grafts extension in a diseased artery, *i.e.*, it was not possible to observe if the largest diameter of the extensions is associated with increased vessel dilation as a consequence.

## CONCLUSION

Treatment with bifurcated bell-bottom stent grafts to repair infrarenal AAAs with associated CIA dilation is a good therapeutic option to preserve hypogastric flow, however, when 38 patients were treated with this method, 17.6% developed endoleak and 15.7% required a reoperation. In addition, this technique can lead to aneurysm enlargement after repair in 35.3% of cases. Artery dilation compared to pretreatment size was, on average, 16% within the first year, 29% during the second year, 57% at the third year, and 95% as of the fourth year, postoperatively.

**Table:** 

Authors' roles & responsibilities		
GJPT	Conception and design study; realization of operations and/or trials; analysis and/or data interpretation; manuscript redaction or critical review of its content; final manuscript approval	
ARF	Conception and design study; realization of operations and/or trials; analysis and/or data interpretation; final manuscript approval	
WKK	Conception and design study; analysis and/or data interpretation; final manuscript approval	
PFS	Analysis and/or data interpretation; manuscript redaction or critical review of its content; final manuscript approval	
KRS	Statistical analysis; manuscript redaction or critical review of its content; final manuscript approval	
JHP	Conception and design study; realization of operations and/or trials; final manuscript approval	
LCDS**	Conception and design study; realization of operations and/or trials; final manuscript approval	
RAC	Conception and design study; analysis and/or data interpretation; manuscript redaction or critical review of its content; final manuscript approval	
